# Understanding chemsex: clinical features, risk constellations and implications for mental health services

**DOI:** 10.1192/j.eurpsy.2026.10370

**Published:** 2026-07-31

**Authors:** M. Gertzen

**Affiliations:** Department of Psychiatry, Psychotherapy and Psychosomatics, Faculty of Medicine, University of Augsburg, Augsburg, Germany

## Abstract

**Abstract:**

Chemsex describes the intentional use of psychoactive substances to facilitate or enhance sexual experiences, most commonly reported among men who have sex with men (MSM) in urban settings. Substances frequently involved include methamphetamine, γ-hydroxybutyrate/γ-butyrolactone (GHB/GBL), mephedrone and other synthetic cathinones, often used in combination and in prolonged sexual sessions. Digital platforms have facilitated network formation and access to drugs, contributing to the expansion and normalization of the phenomenon in certain subcultures. While chemsex may be perceived by participants as enhancing intimacy, pleasure and connectedness, it is increasingly recognized as a complex biopsychosocial issue with significant psychiatric and somatic implications.

From a clinical perspective, chemsex is associated with elevated rates of substance use disorders, mood and anxiety disorders, trauma-related symptoms and personality vulnerabilities. Acute psychiatric presentations may include intoxication syndromes, agitation, psychosis, suicidal ideation and impaired judgment. Repeated use, particularly of stimulants and GHB/GBL, carries risks of dependence, withdrawal syndromes, cognitive impairment and persistent affective instability. Polysubstance use and sexualized drug injection (“slamming”) further increase the likelihood of overdose, blood-borne infections and other medical complications. High-risk sexual behaviors contribute to increased transmission of HIV and other sexually transmitted infections, despite advances in preventive strategies such as PrEP.

Clinicians face specific challenges, including stigma, underreporting, fragmented care between addiction, sexual health and mental health services, and the need for culturally competent, non-judgmental approaches. Routine assessment should integrate sexual health, substance use patterns, psychiatric comorbidity and psychosocial stressors. Emerging perspectives emphasize harm reduction, community-based interventions, trauma-informed care and integrated service models that bridge psychiatry, addiction medicine and infectious disease care.

Chemsex represents a paradigmatic interface between sexuality, substance use and mental health. A nuanced understanding of its motivations, risks and clinical trajectories is essential for developing tailored prevention and treatment strategies within contemporary European psychiatric practice.

**Disclosure of Interest:**

None Declared

